# A Novel Mutation in *CLCN1* Associated with Feline Myotonia Congenita

**DOI:** 10.1371/journal.pone.0109926

**Published:** 2014-10-30

**Authors:** Barbara Gandolfi, Rob J. Daniel, Dennis P. O'Brien, Ling T. Guo, Melanie D. Youngs, Stacey B. Leach, Boyd R. Jones, G. Diane Shelton, Leslie A. Lyons

**Affiliations:** 1 Department of Veterinary Medicine and Surgery, School of Veterinary Medicine, University of Missouri – Columbia, Columbia, Missouri, United States of America; 2 Department of Pathology, University of California San Diego, La Jolla, California, United States of America; 3 Winnipeg Humane Society, Winnipeg, Manitoba, Canada; 4 Institute of Veterinary, Animal and Biomedical Sciences, Massey University, Palmerston North, New Zealand; University of Valencia, Spain

## Abstract

Myotonia congenita (MC) is a skeletal muscle channelopathy characterized by inability of the muscle to relax following voluntary contraction. Worldwide population prevalence in humans is 1∶100,000. Studies in mice, dogs, humans and goats confirmed myotonia associated with functional defects in chloride channels and mutations in a skeletal muscle chloride channel (*CLCN1*). *CLCN1* encodes for the most abundant chloride channel in the skeletal muscle cell membrane. Five random bred cats from Winnipeg, Canada with MC were examined. All cats had a protruding tongue, limited range of jaw motion and drooling with prominent neck and proximal limb musculature. All cats had blepharospasm upon palpebral reflex testing and a short-strided gait. Electromyograms demonstrated myotonic discharges at a mean frequency of 300 Hz resembling the sound of a ‘swarm of bees’. Muscle histopathology showed hypertrophy of all fiber types. Direct sequencing of *CLCN1* revealed a mutation disrupting a donor splice site downstream of exon 16 in only the affected cats. *In vitro* translation of the mutated protein predicted a premature truncation and partial lack of the highly conserved CBS1 (cystathionine β-synthase) domain critical for ion transport activity and one dimerization domain pivotal in channel formation. Genetic screening of the Winnipeg random bred population of the cats' origin identified carriers of the mutation. A genetic test for population screening is now available and carrier cats from the feral population can be identified.

## Introduction

Myotonia is defined as delayed relaxation of voluntarily or reflexively contracted muscle [1] and is due to repetitively firing muscle action potentials after a single stimulus. Individuals affected with various forms of myotonia typically describe a painless, muscle stiffness that remits with several repetitions of the same movement; the so-called ‘warm-up’ phenomenon [2], [3]. In some cases, however, the myotonia does not begin until one or two sets of the same movement are completed, termed delayed myotonia. If symptoms worsen with repeated exercise, paradoxical myotonia is more appropriate, which is usually due to mutant sarcolemmal Na^+^ channels [4].

In humans, myotonia congenita (MC) is characterized by mutations within *CLCN1, a* gene [5] encoding the skeletal muscle voltage-gated chloride channel ClC-1. This chloride channel is responsible for up to 80% of the resting sarcolemmal conductance [4], [6], [7] and belongs to the ClC family of anion channels (ClC-0, ClC-1, ClC-2 and ClC-Ka/Kb) and anion/proton antiporters (ClC-3–ClC-7) [8]. The channel architecture results in two largely independent protopores of ClC channels that are opened and closed by two structurally distinct gating processes [9]. In humans, *CLCN1* is organized into 23 exons [10] and each allelic gene product is thought to contribute to its own pore. The structure of the chloride channel protein was elucidated with electron microscopy [11] and X-ray crystallography [12], [13] following the discovery of bacterial homologues from *Escherichia coli* and *Salmonella typhimurium*. The ClC dimer consists of a transmembrane (TM) and a cytosolic cystathionine beta-synthase (CBS) domain comprising 23 alpha-helices (A-V) and 5 beta-strands. *CLCN1* functions as a homodimer with four proposed sites of dimerization [12], [14].

MC shows autosomal dominant or recessive patterns of inheritance with varying degrees of severity. A correlation between the severity of the phenotype and the mode of inheritance has been suggested; the autosomal dominant (Thomsen Disease, OMIM 160800) form associated with a milder phenotype and the recessive form (Becker Disease or recessive generalized myotonia, OMIM 255700) associated with severe myotonia [15], [16]. The disease phenotype is evident when the net chloride channel conductance is below 50% of normal, thus usually dominant-negative interactions that disrupt the channel are more likely associated with autosomal dominant pattern of inheritance [2], [3]. Mutations that cause early truncations of the protein are thought to present as an autosomal recessive mode of inheritance, due to the loss of the ability to form dimers [17], [18].

In veterinary medicine, MC has been described in several mammalian species. The goat provided the first opportunity to define the cellular defect in myotonic muscle [19], [20], as muscle fibers from myotonic goats exhibited greatly diminished chloride conductance [21]. In this first molecular model, Beck *et al*. (1996) reported a missense mutation affecting the carboxy terminus of *CLCN1* that reduces the channel open probability at physiologic voltages. In goats, MC is inherited as an autosomal dominant trait [22]. Since the first description of caprine MC [23], historical, clinical, electrodiagnostic and histopathologic features have become well-established in horse [24], dog [25], [26] and cat models [27]–[30]. In the feline model, historical and clinical findings, along with routine blood testing, muscle percussion, cardiac examination, electrodiagnostic testing and muscle histopathology have been characterized in two related kittens and in four kittens from separate litters [21], [23]. Both reports speculated that the disease was inherited as an autosomal recessive trait.

In the present study, feline MC from a feral random bred colony was characterized with respect to routine clinical investigations, skeletal muscle histology and immunohistochemistry, and electrodiagnostic findings. Direct sequencing of the candidate gene, *CLCN1*, and analysis of mRNA from muscle biopsies were conducted in the cats.

## Results

### Clinical presentation

**Figure 1:**
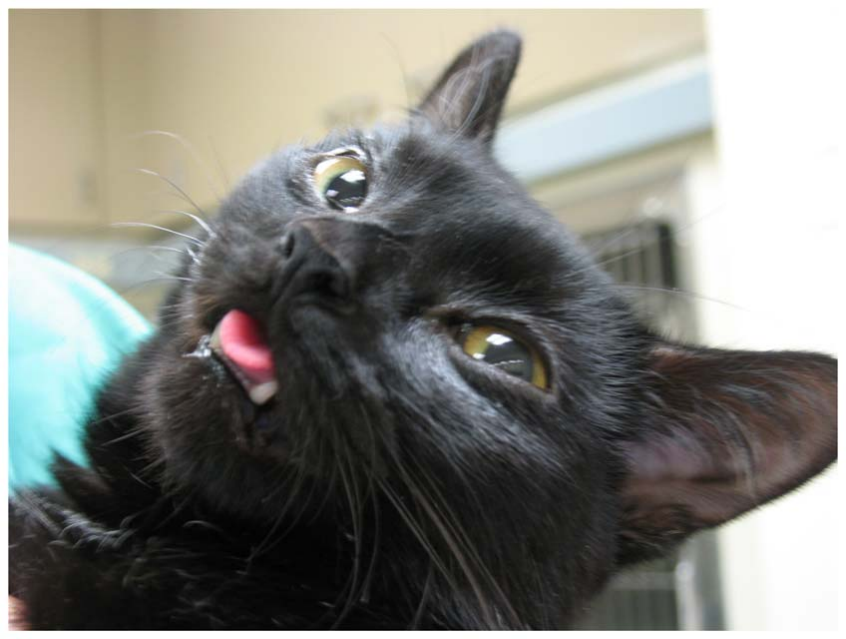
The hypertrophic tongue in a cat affected with myotonia congenita. The tongue is very enlarged and constantly protrudes from the mouth.

Abnormalities on general physical examination common to all five affected cats included a restricted jaw opening, halitosis, varying degrees of gingivitis, pseudoptyalism, marked dental calculus accumulation with palpable loose teeth and evidence of poor grooming habits. Severe muscle hypertrophy was found along the cervical spine, proximal aspects of all limbs (Video S1) and the tongue, which protruded from the mouth (Figure 1 and Video S2). One cat showed signs of respiratory distress (stridor, open mouth breathing) upon examination that lasted for several minutes. Serum creatine kinase (CK) activity was mildly elevated in only one of the five cats tested.

All affected cats had a normal mentation. On cranial nerve examination, abnormalities were limited to blepharospasm after testing both the menace responses and the palpebral reflexes (Video S2). The gait was stiff and short-strided in all limbs, especially in the pelvic limbs, accompanied by a decreased ability to adduct all limbs while walking. While ambulating, four cats would intermittently assume a frozen posture in whatever degree of flexion and/or extension the joints were in at the given instant, and would remain static for several seconds before resuming ambulation (Video S3). No obvious ataxia was observed. In one cat, a plantigrade stance was obvious. Upon postural reaction testing, proprioceptive positioning and hopping were normal in all limbs. Upon visual placing in the thoracic limbs, all cats showed a progressive worsening with this testing procedure, ultimately ending after several repetitions with absent visual placing in the thoracic limbs and active cervical ventroflexion. Abnormalities on spinal reflexes were limited to absent cutaneous trunci reflexes bilaterally in all cats. Percussion of the triceps muscle in one cat produced a prolonged dimpling of the muscle (Video S1). A startle response was not observed in these cats.

### Electromyography, Nerve Conduction Velocity, Repetitive Nerve Stimulation, Muscle Biopsy and Cardiac Findings

**Figure 2:**
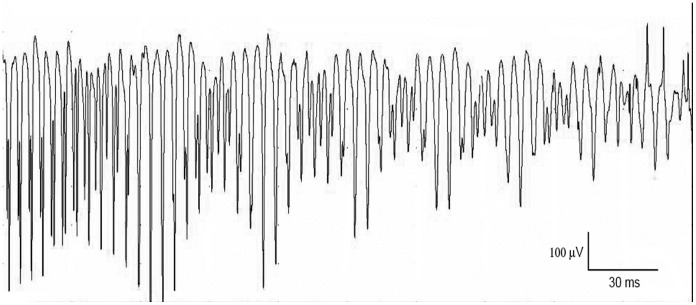
Myotonic discharges in a cat with myotonia congenita. EMG recorded from the biceps femoris muscle showed a sustained run of initially positive biphasic to triphasic spikes with a firing frequency of 240 Hz. Note also the waxing and waning in amplitude of the spike train.

Appendicular as well as axial skeletal muscle was tested. Prolonged insertional activity with myotonic discharges were identified in all skeletal muscles tested and showed the characteristic waxing and waning amplitude (Figure 2 and Video S4). The discharges had a mean amplitude of 277 µV (130 µV, 240 µV and 470 µV) and a mean frequency of 300 Hz (240 Hz, 260 Hz and 400 Hz). Because of the remarkably high frequency of the myotonic discharges, the sound generated from these discharges on the EMG loudspeaker resembled a swarm of bees.

**Figure 3:**
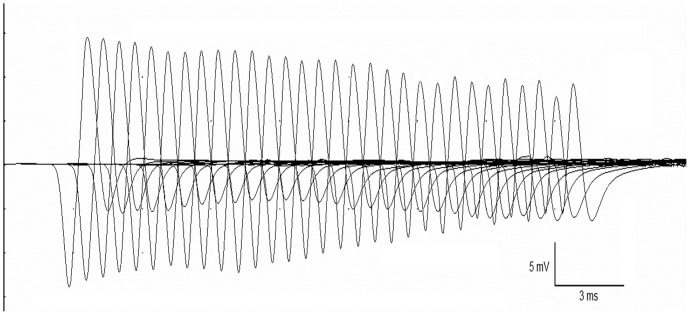
Supramaximal repetitive nerve stimulation in a cat with myotonia congenita. Stimulation of the right tibial nerve at 3 Hz shows a decrement of 48.3% between the 1st and 30th waveforms.

Left tibial nerve motor conduction velocity was tested in two cats and found to be within normal limits. Supramaximal repetitive nerve stimulation was performed at the left distal hock in three cats at a repetition rate of 3 Hz. Decrement was observed in two cats tested, with percentage decreases in area under the curve between the 1^st^ and 10^th^ waveforms of 31.4% and 66.9% and percentage decreases of 48% and 67% between the 1^st^ and 30^th^ waveforms (Figure 3).

**Figure 4:**
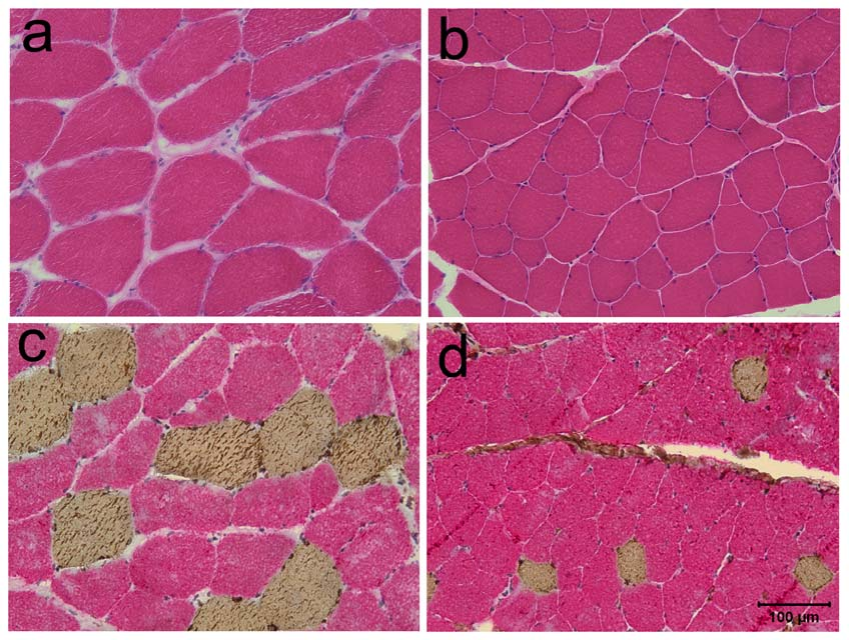
Pathological changes were limited to myofiber hypertrophy in cats with myotonia congenita. a. H&E stained cryosections of the biceps femoris muscle. Hypertrophy involved both type 1 and type 2 fibers. b. H&E stain of biceps femoris muscle. c. Antibody staining for type 1 (brown color) and type 2 (red color) fibers. For comparison of fiber size, an unaffected approximately age matched control cat muscle is shown. b. H&E stain of biceps femoris muscle; d; antibody staining for type 1 and type 2 fibers. Magnification bar  = 100 µm for all images.

Myofiber size ranged from 50–150 µm (normal feline reference interval 40–50 µm) [31]. A normal mosaic pattern of muscle fiber types was present. Intramuscular nerve branches were normal in appearance. No inflammation, necrosis, fibrosis or other cytoarchitectural abnormalities were found (Figure 4).

Cardiac auscultation of all cats was normal. Thoracic radiographs were taken in two cats and echocardiograms were performed in four cats. Thoracic radiographs showed no evidence of heart enlargement or congestive heart failure. Two-dimensional echocardiography revealed several moderator bands in the left ventricle in all four cats evaluated. Left ventricular diastolic function was normal in all cats evaluated based on transmitral flow patterns, isovolumic relaxation times, and/or tissue Doppler mitral annular motion. No cardiac hypertrophy or chamber enlargement was noted in any of the cats evaluated. Atrial premature complexes of unknown origin were noted in one cat.

### *CLCL1* sequence analysis and genotyping

**Figure 5:**
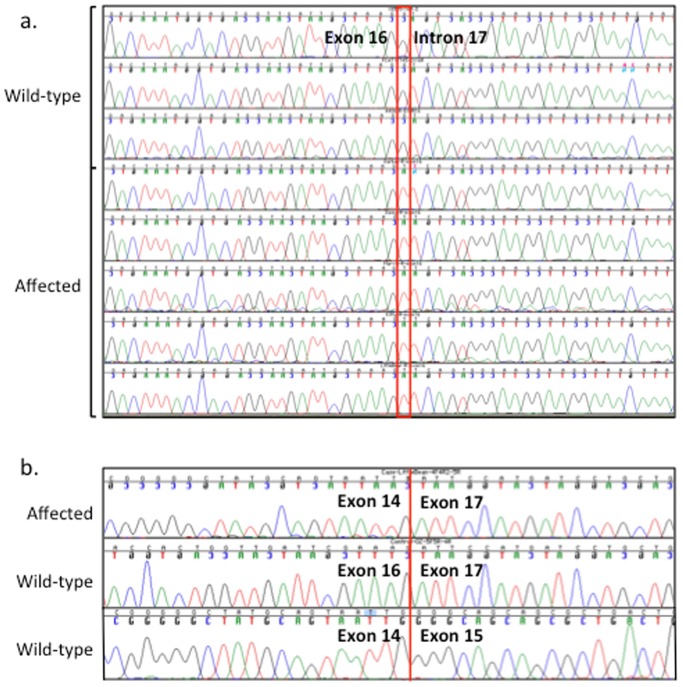
Electropherogram representing the genomic and RNA sequence of *CLCN1* in wild-type and myotonia congenita cats. a. Genomic sequence of three control cats and the five affected cats. The red rectangle indicates the c.1930+1G>T polymorphism in the first base of intron 16 associated with MC in the domestic cat. b. RNA sequence lacks exon 15 and 16, while no alterations are observed in the control cat at exon junctions 14–15 and 16–17.

**Figure 6:**
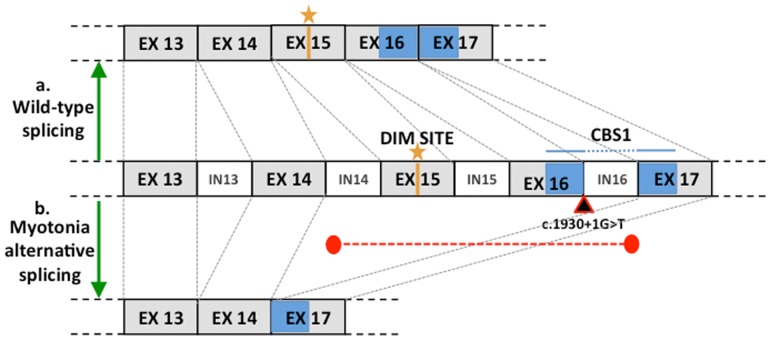
Schematic representation of *CLCN1* splicing in the wild-type cat and myotonia congenita cats. In exon 15, the yellow line represents the third dimerization site (dim site), while the blue box represents the CBS1 domain, present in exon 16 and 17. The black triangle represents the identified mutation and the two red circles connected by a red dashed line represent the donor splice site and the acceptor splice site in the mutated protein. a. Normal splicing occurring in the wild-type subject, both dimerization sites and CBS1 are present. b. In the affected cat, the mutation in intron 16 is associated with the absence of exons 15 and 16, and, therefore the protein lack the dimerization domain.

The entire *CLCN1* coding sequence (GeneBank accession no. KJ561451) and partial 5′ and 3′ UTRs were analyzed for six cats (Table 1) representing the five affected cats and one random bred control. In humans, *CLCN1* has one isoform and the length of the coding region within the transcript is 2967 bp. In the cat, *CLCN1* has 23 exons (Table S1), the boundaries were confirmed by genomic sequencing of the five cats used for the analyses of *CLCN1*, producing a 2,970 bp coding sequence (CDS) that translates into 989 amino acids (Figure S1). The average CDS identity between humans and cats is 87.5% and at the protein level, *CLCN1* in felines is 89.5% identical to humans. Seven of the seventeen identified polymorphisms are exonic mutations (Table 1). Two mutations in exon 18 result in an amino acid substitutions, however, they were not concordant with the disease phenotype. One of the ten intronic mutations segregated concordantly with the phenotype and affects an exon/intron splice site in affected cats. The c.1930+1G>T transversion altered the 5′ splice site at the junction of exon 16 and intron 16 (Figure 5a). The effect of this polymorphism was investigated at the RNA level (Figure 5b). All the affected cats showed the same haplotype across all the identified polymorphisms (Table 1). All the mutations were confirmed by the RNA transcript sequence obtained from muscle tissues of affected and wildtype cats. Interestingly, the identified mutation is associated with the upstream absence of exons 15 and 16 (Figure 6). Intron 14 sequence showed polymorphisms in the control cat only, in a heterozygous state, while the affected cat sequence was identical to the wildtype sequence. To exclude retrotranscription artifacts, the RNA experiment was conducted twice, beginning at the RNA isolation step. *In silico* translation of the altered transcript predicts the lack of 116 amino acids, from residues 557 to residue 643 of the protein (Figure S1). The predicted premature *CLCN1* protein truncation would cause the partial absence of the first highly conserved CBS1 (cystathionine β-synthase) domain and the third dimerization domain p.578Y. The strength of the 5′ splicing sites (5′ss) was calculated for exon-intron 14, 15 and 17 boundaries using the Maximum Entropy Model score, the Maximum Dependence Decomposition Model score, the first-order Markov Model and the Weight Matrix Model score. The strength of the 5′ss in all the tested boundaries was similar in each method when compared, suggesting exon-intron 15 5′ss as the strongest. Complete donor splice site strength is confirmed in the mutated sequence (Table S2).

**Table 1:** SNPs analyses of *CLCN1* in cats with and without myotonia congenita.

Genotype																	
Sample ID	Pheno	5′UTRc.-174A>G	5′UTRC.1-38insC	I3c.433+451G>A	E4c.558C>T	E5c.567T>C	I5 c.696+72C>T	I8 c.97+33 T>C	I11 c.1251+190delTGTTTGTT	E12c.1278C>G	E15c.1668C>T	I16*c.1930+1G>T	I17c.2175-40A>G	E18c.2190C>G	E18c.2216T>C	I22c.2598-65C>T	E23c.2631C>T
Cat1	Affected	G	insC	-	T	T	T	C	Deletion	G	T	T	G	C	T	T	T
Cat2	Affected	G	insC	A	T	T	T	C	Deletion	G	T	T	G	C	T	T	T
Cat3	Affected	G	insC	A	T	T	T	C	Deletion	G	T	T	-	C	T	T	T
Cat4	Affected	G	insC	-	T	T	T	C	Deletion	G	T	T	-	C	T	T	T
Cat5	Affected	-	insC	A	T	T	T	C	Deletion	G	T	T	-	C	T	T	T
Control	Wildtype	A	WT	G	T	T	C	T	Wildtype	C	C	C	-	C	T	C	C
Ensembl	Wildtype	A	WT	-	C	C	C	C	Wildtype	C	C	C	A	G	C	T	C
AA change					-	-				-	-			P>A	L>P		-

*This polymorphism is investigated in detail by the authors.

Within 244 cats screened for the c.1930+1G>T polymorphism (Table 2), the mutation was only found to be heterozygous in three cats from the Winnipeg feral population (n = 35). The cats pertaining to the 26 breeds (n = 182) as well as the two random bred populations (n = 14) all tested wildtype.

**Table 2:** Domestic cats genotyped for the *CLCN1* mutation associated with myotonia congenita.

				Genotype	
Breed	Phenotype	No.	G/G	G/T	T/T
Abyssinian	Normal	9	9	0	0
American shorthair	Normal	8	8	0	0
Bengal	Normal	7	7	0	0
Birman	Normal	7	7	0	0
British shorthair	Normal	7	7	0	0
Burmese	Normal	7	7	0	0
Chartreux	Normal	7	7	0	0
Cornish Rex	Normal	8	8	0	0
Devon Rex	Normal	7	7	0	0
Egyptian Mau	Normal	7	7	0	0
Japanese Bobtail	Normal	7	7	0	0
Korat	Normal	7	7	0	0
Maine Coon	Normal	9	9	0	0
Manx	Normal	7	7	0	0
Norwegian Forest Cat	Normal	9	9	0	0
Ocicat	Normal	7	7	0	0
Oriental	Normal	7	7	0	0
Persian	Normal	7	7	0	0
Ragdoll	Normal	7	7	0	0
Russian Blue	Normal	7	7	0	0
Siamese	Normal	7	7	0	0
Siberian	Normal	7	7	0	0
Sphynx	Normal	7	7	0	0
Tonkinese	Normal	7	7	0	0
Turkish Angora	Normal	7	7	0	0
Turkish Van	Normal	7	7	0	0
Random Bred (Winnipeg)	Affected	5	0	0	5*
	Normal	35	32	3	0
Random Bred (France, USA)	Normal	14	14	0	0
Total		244	236	3	5

*Cats genotyped by direct sequencing.

### Immunohistochemical localization of voltage-dependent chloride (Cl^-^) channels

**Figure 7:**
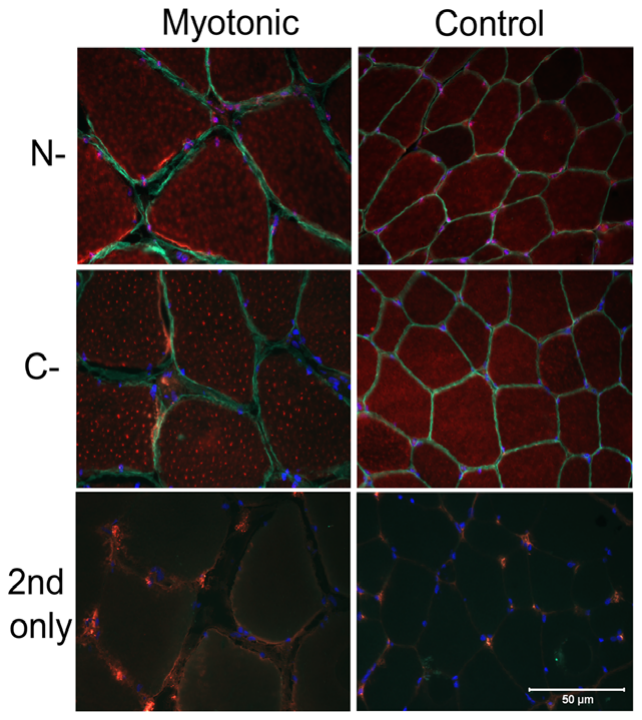
Immunofluorescent staining for localization of antibodies against the N-terminus and C-terminus of *CLCL1*. Cryosections of biceps femoris muscle from a representative myotonic cat and approximately age matched control. A similar pattern of punctate cytoplasmic staining was found with both antibodies in the myotonic and control cat (rhodamine filter, red color). An antibody against dystrophin was used to localize the muscle sarcolemma (FITC, green color). Bar  = 50 µm for all images.

**Figure 8:**
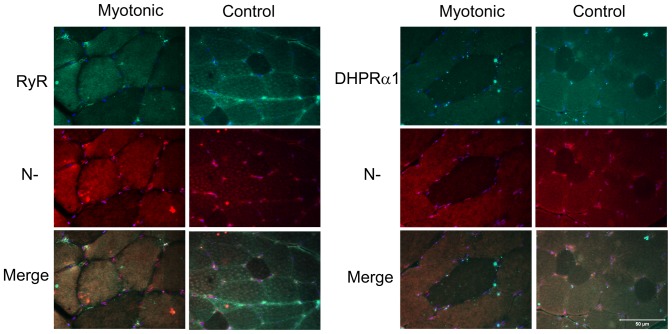
Antibody markers against RYR, DHRPα1 and *CLCN1*. To further investigate the subcellular localization of the chloride channels in myotonic cats, antibody markers for the sarcoplasmic reticulum (RYR; ryanodine receptor, green) and T-tubule system (DHRPα1, green) were co-stained with antibodies against *CLCL1* (red) and compared to control muscle. The merge figures for both myotonic and control cats (yellow) show close proximity of CLC1 to the sarcoplasmic reticulum and T-tubule system. Bar  = 50 µm for all images.

Cryosections (8 µm) from the biceps femoris muscle of affected and control cats were incubated with rabbit polyclonal antibodies against the C-terminus and N-terminus of *CLCN1* and co-stained with a monoclonal antibody against dystrophin (marker for the muscle sarcolemma). The sections were then processed by indirect immunofluorescence. Staining using both antibodies against *CLCN1* was localized to the interior of the myofiber and was distinctly punctate in nature without co-localization with dystrophin to the muscle sarcolemma (Figure 7). To further investigate the subcellular localization of the chloride channels, antibody markers for the sarcoplasmic reticulum (ryanodine receptor) and T-tubule system (dihydropyridine receptor DHPRα1) were co-stained with both antibodies against *CLCN1* and compared to control muscle. Staining strongly suggested either co-localization or a close association with the T-tubule system (Figure 8).

## Discussion

Voltage-dependent chloride (Cl^-^) channels are transmembrane proteins that represent Cl^-^ permeation pores. Chloride channels are as abundant as cation channels (Na^+^, K^+^, Ca^2+^) and participate in many physiological tasks, including the maintenance of normal cellular excitability, the control of neurotransmitter release and the transport of ions across epithelial cells. MC was one of the first genetic diseases shown to be caused by mutations in genes encoding ion channels [5], [32]. The aim of this paper was to describe the clinical characteristics of a cohort of cats with feline MC along with novel electrophysiologic findings and the molecular characterization of the mutation within the *CLCN1* gene.

MC was characterized previously in a 5-month old intact male and in 9-month old intact female domestic shorthair littermates [28], as well as four closely related kittens from 2 related queens and unknown sires [29]. Although a mutation was not identified, both reports suggested the condition was a hereditary disease. Findings in common to both studies included an unthrifty appearance, a short-strided, choppy gait (more severe in the pelvic limbs), blepharospasm upon palpebral reflex testing or startling, dysphonia and prolonged dimpling of the tongue after percussion (percussion myotonia). The general physical examination and neurologic findings in the cats of this study are consistent with both previous reports. All cats from this feral breeding colony showed a protruding tongue with excessive drooling, prominent neck and proximal limb musculature, decreased range of jaw motion, oral disease, bilateral blepharospasm upon testing, a short-strided gait with decreased ability to adduct the limbs and a progressive decrease through successive attempts of visual placing in the thoracic limbs. This progressive decrease in the visual placing response was ascribed to transient weakness and no clinical significance was ascribed to the absent cutaneous trunci reflexes as this reflex has been previously shown to be sometimes absent in otherwise neurologically normal cats [62]. No clinical evidence of cardiac disease was detected in this study, consistent with previous reports of feline MC [28]. MC is a disease that typically does not affect either smooth or cardiac muscle [33].

Previously reported abnormal electromyographic findings in feline MC included increased insertional activity and myotonic discharges, with no evidence of defective nerve conduction. These findings were duplicated in all cats tested in the present study. In addition, the frequency of myotonic discharges reported herein was unique, with a mean frequency of 300 Hz. To our knowledge, this is the first report documenting such a high frequency of myotonic discharges. Classically, myotonic discharges occur with a frequency of 50–100 Hz and are described as sounding like a motor cycle revving or a dive bomber [1]. The higher frequency of myotonic discharges in the cat is likely the reason for the atypical sound in these cats, resembling a ‘swarm of bees’. Myotonic discharges that wax and wane are the characteristic electrophysiologic abnormality in MC. Diminished chloride conductance across the sarcolemma leads to increased input resistance such that a smaller membrane depolarization is more likely to trigger an action potential. The restricted extracellular space within the transverse tubule system in the muscle leads to an accumulation of K+ ions following action potentials. Reduced chloride conductance in affected individuals increases the likelihood of spontaneous after-depolarizations that can eventually reach the sarcolemmal threshold voltage for action potential initiation [7].

The cats tested herein with repetitive nerve stimulation at 3 Hz showed pathologic decrement, a reproducible decline in the area under the curve of the compound muscle action potential (CMAP). The stimulation frequency of 3 Hz was chosen within this study based on higher frequencies of stimulation showing physiologic CMAP decremental responses in dogs [60], [61]. Decrement can be an electrophysiologic feature of myotonia congenita in some cases that represents the neurophysiologic counterpart of the initial weakness that patients often ‘warm-up’ out of [34]. Although not a classical finding in all cases of MC, this is not the first instance of pathologic decrement in veterinary patients with MC, as this has been reported previously in the Chow Chow dog [58]. This shared feature of pathologic decrement between the cats presented herein and these Chow Chow dogs suggests the possibility of a common molecular pathophysiology, although the molecular basis of MC in the Chow Chow will need to be addressed in order to explore this possibility.

The decrement in MC may ultimately result from the prolonged after-depolarization of the sarcolemmal membrane, secondary to potassium accumulation in the transverse tubules [39]. It has been proposed that the decrement is the electrophysiologic correlate of weakness in some forms of MC. The weakness may be attributed to muscle hypoexcitability secondary to sodium channel inactivation (depolarization block) at times of more extreme muscle depolarization, with motor axons subsequently firing at higher rates to control a weak muscle [59]. The degree of decrement, however, has not been shown to be associated with clinical severity [35]. A recent cohort study in humans has shown that decrement with the 3 Hz nerve stimulation protocol had a sensitivity of 66% for recessive MC with good reproducibility, whereas those patients with dominant MC or myotonia secondary to a defect affecting the skeletal muscle sodium channel showed no decrement at such a stimulation frequency. Decrement in recessive MC has been suggested to be related to variation in the *CLCN1* alleles, including nonsense, splice site frameshift and missense mutations [35]–[38].

In people with MC, non-specific histologic changes include abnormal variations in myocyte diameter, fiber hypertrophy, absence of type IIb fibers, central location of fiber nuclei and mitochondrial aggregates [40]. Variable findings have been described for both light and electron microscopy in feline MC, including occasional degeneration of individual myofibers associated with localized proliferation of sarcolemmal nuclei with few centrally located nuclei, moderate diffuse myofibril hypertrophy, rounding of the sarcoplasm, clear cytoplasmic vacuoles within many myofibers and mild dilatation of transverse T-tubules [27]–[29]. The mean myofiber perimeter and areas were previously measured and compared to age-matched controls using image software, showing increased perimeters and areas of myofibers in affected cats [27], [28]. The muscle profiles in this study showed hypertrophy of both type 1 and type 2 fibers with a normal mosaic pattern of muscle fiber types and no evidence of inflammation, necrosis, fibrosis or other specific cytoarchitectural abnormalities, consistent with a diagnosis of MC.

In our study, results of immunoflourescent staining in both affected and control cats support localization of the chloride channels close to or at the location of the T-tubules. Immunofluorescent staining for chloride channels in both affected and control cat muscle did not support localization to the sarcolemma. Since staining was similar in both affected and control cats, the mutation identified in these myotonic cats likely results in impaired ion channel function rather than a mislocalization. However, additional more sophisticated studies that are beyond the scope of this study would be necessary to determine channel density or more precise localization.

In recent years, a large number of mutations associated with MC have been identified in *CLCN1* in dog [41], [42], goat [22], horse [43], water buffalo [44], mouse, [45] and human [5]. These mutations are dispersed throughout the entire coding and non-coding regions and result in distinct alterations of channel kinetics. Unexpected insights into the structure-function relationship are provided by the discovery of random sequence alterations that impair channel function, providing an alternative approach to identify elements in the sequence essential in channel function. The chloride channel consists of transmembrane domains, three dimerization domains and cystathionine β-synthase domains, critical for ion transport activity.

In the current study, *CLCN1* was analyzed in five cats with classical MC. Relatedness within the cats included in the study is not known, however, the cats were identified as originating from the same feral colony in Winnipeg, Canada. An intronic mutation in intron 16 leads to a splicing error, confirmed by the cDNA analysis, which translates to a lack of exon 15 and 16 and a 116 amino acids gap in the protein. Since the alternative splicing is atypical, the experiment was conducted from freshly isolated RNA and retrotranscription products. The same splicing variation was detected in both experiments, excluding retrotranscription artifacts as being responsible for the observed splicing. The entire sequence of intron 14 was also acquired and excluded possible intronic mutation associated with the detected splicing variant. The c.1930+1G>T appears to be the polymorphism associated with MC in the domestic cat.

Splice sites (5′ss) are the elements at the 5′ end of introns and are extremely diverse, as revealed by the thousands of different sequences that act as 5′ss in the human transcriptome [46]. Most of the 5′ss are recognized by base-pairing with the 5′ end of a small nuclear RNA (snRNA) called U1 [47], [48]. To date, over 9000 sequence variants in the −3 to +6 region of the 5′ss are recognized in humans as 5′ss consensus [46]. The use or avoidance of 5′ss could depend on other sites and is not necessarily an intrinsic property of any given sequence, as in the case of some sequences in β-globin where sequences that resemble 5′ss were used when a natural 5′ss was inactivated [49], [50]. The mechanism is termed cryptic 5′ss. Previous work had shown that a mutation within a 5′ss might inactivate it or lead to the use of cryptic 5′ss [51]. In the present work, an unusual alternative splicing that leads to the lack of two exons upstream of the mutation is presented. The 5′ss strength analysis results exclude a significant difference in the strength of exon-intron 14 boundaries, excluding a gain of strength or the presence of a strong 5′ss as possible mechanisms involved in exon 15 and 16 skipping. Moreover, the complete sequence of intron 14 reveals no mutations concordant with the phenotype and possibly responsible for the alternative splicing. A second hypothesis is that entire exons can be skipped when located in an internal RNA loop [52]. This evidence is supported in numerous studies where secondary structures showed influence in 5′ss selection and subsequent splicing [53]. A striking example is provided by the *MAPT* exon 10 alternative splicing, whose inclusion levels are dictated by the efficiency of the 5′ss recognition, which is compromised by downstream intronic nucleotides responsible for a base-pairing structure [54]. The mutation occurring in intron 16 could be associated with the appearance of a pre-mRNA structure, such as loop, during the splicing event directly responsible for the lack of both exons 15 and 16, or indirectly affecting the performance of RNA-binding proteins and the rate of transcription.

The characterized alteration in *CLCN1* is predicted to cause a lack of 116 amino acids in the protein, from position 557 to position 643. The alteration deletes part of the first CBS domain [55]. Mutations within the domain, critical for ion transport activity, are associated with recessive and dominant forms of MC [56]. Moreover, the deletion removes the third, highly conserved, dimerization domain. It is possible to hypothesize that the lack of one of the dimerization sites prevents the mutated allelic gene product from being incorporated in the dimer, although the mutated product is still incorporated into the membrane. A recessive mode of inheritance was also confirmed by the genotyping results, where the heterozygous cats were confirmed to be healthy. Carrier cats were only identified in the Winnipeg feral population, supporting the hypothesis that a novel mutation that arose in an intact feral cat and was spread amongst the local feline random bred population. A genetic test is now available and carrier individuals can be identified and sterilized to prevent further spread of the genetic alteration within the local population.

Myotonia congenita is one of numerous inherited diseases of skeletal muscle that have been defined at the molecular level in recent years and this disease model had provided cogent insights regarding muscle excitation and contraction. For the first time, MC in *Felis silvestris catus* was molecularly defined and associated with an early truncation of the *CLCN1* protein, establishing the cat as a potentially valuable new animal model of this disease.

## Materials and Methods

### Sample collection

Private owners of affected and control cats were recruited to voluntarily participate in the study. All procedures were approved by the University of Missouri, Animal Care and Use Committee. Muscle biopsy samples were obtained from a privately owned cat after acquiring informed owner consent and done for routine diagnosis. Five affected cats privately owned were obtained from the same cat shelter in Winnipeg, Canada. The shelter obtained these cats from various sources, including local feral populations under population control. DNA samples from the Canadian feral cat population and from the control populations (26 breeds and random bred cats from US and around the world) were collected from the cats non-invasively using buccal swabs at cat shows, shelters and the owners’ homes (Table 2). DNA was isolated from buccal swabs using the DNAeasy Kit (Qiagen).

### Clinical description and Sampling

The five affected adult cats from Canada were brought to the Neurology service at the University of Missouri – Columbia, Veterinary Medicine Teaching Hospital for examination and diagnostic testing. A history of all cats was collected from the veterinarian who referred cats for diagnostic testing. All cats underwent both a general physical examination and a detailed neurologic examination, as well having complete blood counts and serum biochemistry panels. Urinalyses were assayed on two cats. Thoracic radiographs were performed on two cats. An echocardiogram was performed on four cats. All electrodiagnostic testing was performed using a Cadwell Sierra Wave machine (Kennewick, WA) with concentric (coaxial) needle electrodes while the cats were maintained under general gaseous anesthesia. Electromyography, nerve conduction velocities and repetitive nerve stimulations were performed within 10–15 minutes of induction of general anesthesia in three tested cats. Biopsies from the right biceps femoris muscle were collected under general anesthesia in three cats. The muscle biopsy was bisected: the first portion was placed in a saline-dampened gauze sponge followed by submission under refrigeration for histopathologic assessment (Comparative Neuromuscular Laboratory; University of California, San Diego). The second portion was transferred into RNA later (Qiagen, Valencia, CA). RNA was isolated using the Invitrogen RNA mini Kit (Invitrogen, Carlsbad, CA).

### *CLCN1* genomic analysis

The genomic analysis of *CLCN1* was conducted on genomic DNA from seven cats, including, the five affected random bred cases from Winnipeg and two random bred controls available in the laboratory. The complete CDS (23 exons) of *CLCN1* is publicly available (http://ensembl.org) and can be found on cat chromosome A2: 157,284,157–157,315,463 (assembly version Felis_catuts_6.2). *CLCN1* intron 14 was fully amplified using the SequalPrep long range PCR kit (Invitrogen) as per manufacturers recommendations and sequenced using internal primers (Table S1). Primers were designed in both UTRs and intronic regions, flanking the exons. Primers were tested for efficient product amplification on a DNA Engine Gradient Cycler (MJ Research, GMI, Ramsey, MN) and the final PCR magnesium concentrations and annealing temperatures for each primer pair are shown in Table S1. PCR and thermocycling conditions were conducted as previously described [57]. The PCR products were purified with ExoSap (USB, Cleveland, OH) per the manufacturer's recommendations and directly sequenced using the BigDye terminator Sequencing Kit v3.1 (Applied Biosystems, Foster City, CA). Sequences were verified and aligned using the software sequencer version 4.10 (Gene Codes Corp., Ann Arbor, MI).

### *CLCN1* mRNA analysis

Total RNA was extracted using the RNA mini Kit (Invitrogen) from muscle from an affected cat and a control random bred cat. Complementary DNA templates were synthesized using SuperScript III (Invitrogen, Carlsband, CA) by reverse transcription of 1 µg of total RNA with gene specific primers (Table S1) and PolyT to obtain partial 5′ UTR, CDS and partial 3′ UTR. Each cDNA sample was subjected to PCR using primers (10 µM each) combined as follow: F1 - R1, F2 - R2, F3 - R3, F3 - R5, F4 - R4, F4 - R6, F5 - R5, F6 - R6, F6 - R7. The PCR conditions were: 1.5 mM Mg, 2 µl of cDNA in a total volume of 20 µl. The PCR cycle was conducted as previously described [57]. The PCR products with appropriate lengths were purified using the ExoSap (USB) enzyme per manufacturer's recommendations. Purified genomic products were directly sequenced in both directions using BigDye Terminator Sequencing Kit v3.1 (Applied Biosystems) and electrophoretically separated on an ABI 3730 DNA analyzer (Applied Biosystems).

### *CLCN1* 5′ss strength analysis

The strength of the 5′ splicing sites (5′ss) was calculated on nine bases fasta sequences (the last three bases of the exon and the first six bases of the adjacent intron) with the online program MaxEntScan (http://genes.mit.edu/burgelab/maxent/Xmaxentscan_scoreseq.html). The program models short sequence motifs, such as those involved in RNA splicing, which simultaneously accounts for non-adjacent, as well as adjacent, dependencies between positions. This method is based on the 'Maximum Entropy Principle' and generalizes most previous probabilistic models of sequence motifs such as weight matrix models and inhomogeneous Markov models.

### *CLCN1* SNP genotyping

Genotyping was conducted using the High-Resolution Melting (HRM) assay. A real-time PCR was carried out on a Rotor-Gene Q Thermal cycler (Qiagen) and 239 samples from 28 populations were tested. PCR was performed in 10 µl reactions with 10 ng genomic DNA using the type-it HRM PCR kit (Qiagen) in accordance with the manufacturer's instructions with HRM forward and reverse primers (Table S1). The assay was optimized using DNA from five wild-type samples and five affected samples, and DNA concentration was normalized to ∼15 ng/µl for each sample to mimic average sample concentration from buccal swab isolation. Heterozygous samples for the assay optimization were made by combining wild-type and affected DNA with a 1: 1 ratio. Graph of melting curves from the optimization are shown in Figure S2. A two-step cycling protocol consisting of an initial denaturation of 95°C for 5 min followed by 40 cycles of 95°C for 10 s and a combined annealing/extension step at 58°C for 30 s was carried out. The samples were subsequently subjected to a 65°C isotherm step for 90 s followed by a temperature gradient from 65°C to 85°C heating 0.1°C per step with 2 s wait at each step. Each genotype was analyzed in duplicate. The genotype was assigned using the Rotor-Gene ScreenClust HRM Software (Qiagen) in a supervised mode on the normalized melting curve.

### Histopathology, immunohistochemistry and immunofluorescence

Unfixed specimens from the biceps femoris muscle of three cats affected with MC were shipped under refrigeration by an express service to the Comparative Neuromuscular Laboratory, University of California San Diego. Samples were immediately flash frozen in isopentane pre-cooled in liquid nitrogen then stored at −80°C until further processed. Control cat biceps femoris muscle was from the frozen tissue archives of the Comparative Neuromuscular Laboratory. Cryosections (8 µm) in myotonic cats and controls were cut and stained with H&E, or incubated with a mouse monoclonal antibody to myosin heavy chain slow (type 1 fibers, MyHC-slow, Leica Biosystems) or a mouse monoclonal antibody to myosin heavy chain fast (type 2 fibers, MyHC-fast, Leica Biosystems) muscle fibers. Color was developed using either Vector red substrate kit (Vector SK-5100) or peroxidase substrate kit DAB (Vector, SK-4100) as per manufacturers instructions.

For immunofluorescence analysis, cryosections from the biceps femoris muscles of the three myotonic cats and two control cats were fixed in cold acetone/methanol (1∶1) for 5 minutes, and then incubated at 4°C overnight with the following antibodies at appropriate dilutions: mouse anti-RyR antibody (Santa Cruz Biotechnology, 1∶100), mouse anti-DHPR alpha 1 (Abcam, 1∶100), mouse anti-dystrophin rod-domain (Novocastra, NCL-DYS1, 1∶20), rabbit anti-N-terminus of Chloride Channel 1 antibody (antibodies-Online Inc. 1: 250) and rabbit anti-C-terminus of Chloride Channel 1 antibody (antibodies-Online Inc. 1: 250). After washing, the slides were incubated in fluorescein (FITC)-conjugated goat anti-mouse IgG (Jackson ImmunoResearch, 1∶200) or rhodamine red-conjugated goat anti-rabbit IgG (Jackson ImmunoResearch, 1∶200) for 1 hour at room temperature. The sections were mounted in ProLong Gold antifade reagent with Dapi (Invitrogen) and examined under a fluorescent microscope.

## Supporting Information

Figure S1 ***CLCN1*** **protein alignment of** ***Homo sapiens*** **, domestic cat wildtype (wt) and mutated (mut) protein.** In yellow the three amino acids involved in protein dimerization and in green the two CBS domains. The affected cat protein prediction lacks 116 amino acids, from position 557 to position 643 of the protein sequence.
(DOCX)

Figure S2 Normalized melting curve graph of wild-type, homozygous and heterozygous samples for the c.1930+1G>T polymorphism. The graph represents three different melting curves patterns. The cohort with the highest melting temperature represents the wild type samples, the cohort with the lowest melting curve represents the homozygous affected samples. Samples with a melting curve in between represents the heterozygous group.
(DOCX)

Table S1 **Primers to analyze feline** ***CLCN1.***
(DOCX)

Table S2 **5′ss exon-intron boundaries strength.**
(DOCX)

Data S1 ***CLCN1*** **sequences.** *CLCN1* DNA and RNA sequences generated on affected and control cats.
(ZIP)

Video S1 **Muscle hypertrophy and sustained contraction in a cat with myotonia congenita.** The muscle hypertrophy is apparent when the limb was shaved under anesthesia prior to muscle biopsy. Percussion of the muscle with a reflex hammer produces a sustained contraction of the muscle (dimpling).
(MOV)

Video S2 **Blepharospasm and tongue hypertrophy in a cat with myotonia congenita.** Eliciting the palpebral reflex produces a sustained blepharospasm. The tongue is hypertrophied and the cat cannot fit retract the tongue inside his mouth.
(MOV)

Video S3 **Gait of a cat with myotonia congenita.** The cat begins to move with a stiff, short-strided gait that improves with subsequent steps.
(MOV)

Video S4 **EMG of a cat with myotonia congenita under general anesthesia.** The EMG shows increased insertional activity with a high frequency and with a waxing and waning amplitude producing the sound akin to a swarm of bees.
(MOV)
