# Supplementary material for: A Novel Mutation in CLCN1 Associated with Feline Myotonia Congenita
Source: PLoS One. 2014 Oct 30;9(10):e109926. doi: 10.1371/journal.pone.0109926 (PMC4214686; doi:10.1371/journal.pone.0109926)
Supplement: Figure S1 — CLCN1 protein alignment of Homo sapiens , domestic cat wildtype (wt) and mutated (mut) protein. In yellow the three amino acids involved in protein dimerization and in green the two CBS domains. The affected cat protein prediction lacks 116 amino acids, from position 557 to position 643 of the protein sequence. (DOCX) [file pone.0109926.s001.docx]

**Supplementary files:**

1 50

HSap MEQSRSQQRGGEQSWWGSDPQYQYMPFEHCTSYGLPSENGGLQHRLRKDA

WT MERSESQRRGGEQSWWGSAPQYQYMPFEHCTSYGLPSENGALQHRLRRDA

MUT MERSESQRRGGEQSWWGSAPQYQYMPFEHCTSYGLPSENGALQHRLRRDA

51 100

HSap GPRHNVHPTQIYGHHKEQFSDREQDIGMPKKTGSSSTVDSKDEDHYSKCQ

WT GPRPNTRPTQIYGHHKQQPPHKEQGMGMPPKTGSSDSLDSKDEDHSSKCQ

MUT GPRPNTRPTQIYGHHKQQPPHKEQGMGMPPKTGSSDSLDSKDEDHSSKCQ

101 150

HSap DCIHRLGQVVRRKLGEDGIFLVLLGLLMALVSWSMDYVSAKSLQAYKWSY

WT DCMRRLGHVVRRKLGEDWIFLVLLGLLMALVSWSMDYVSAKSLQAYKWTY

MUT DCMRRLGHVVRRKLGEDWIFLVLLGLLMALVSWSMDYVSAKSLQAYKWTY

151 200

HSap AQMQPSLPLQFLVWVTFPLVLILFSALFCHLISPQAVGSGIPEMKTILRG

WT YQMQPSLPLQYLVWVTFPLTLILFSALFCHLISPQAVGSGIPEMKTILRG

MUT YQMQPSLPLQYLVWVTFPLTLILFSALFCHLISPQAVGSGIPEMKTILRG

201 250

HSap VVLKEYLTMKAFVAKVVALTAGLGSGIPVGKEGPFVHIASICAAVLSKFM

WT VILKEYLTLKAFVAKVVALTAGLGSGIPVGKEGPFVHIASICAAVLSKFM

MUT VILKEYLTLKAFVAKVVALTAGLGSGIPVGKEGPFVHIASICAAVLSKFM

251 300

HSap SVFCGVYEQPYYYSDILTVGCAVGVGCCFGTPLGGVLFSIEVTSTYFAVR

WT SMFCGVYEQPYYYTDMLTVGCAVGVGCCFGTPLGGVLFSIEVTSTYFAVR

MUT SMFCGVYEQPYYYTDMLTVGCAVGVGCCFGTPLGGVLFSIEVTSTYFAVR

301 350

HSap NYWRGFFAATFSAFVFRVLAVWNKDAVTITALFRTNFRMDFPFDLKELPA

WT NYWRGFFAATFSAFVFRVLAVWNKDAVTITALFRTNFRMDFPFDLQELPA

MUT NYWRGFFAATFSAFVFRVLAVWNKDAVTITALFRTNFRMDFPFDLQELPA

351 400

HSap FAAIGICCGLLGAVFVYLHRQVMLGVRKHKALSQFLAKHRLLYPGIVTFV

WT FAIIGICCGFLGAVFVYLHRQVMLGVRRHKALSRFLAKHRLLYPGIVTFV

MUT FAIIGICCGFLGAVFVYLHRQVMLGVRRHKALSRFLAKHRLLYPGIVTFV

401 450

HSap IASFTFPPGMGQFMAGELMPREAISTLFDNNTWVKHAGDPESLGQSAVWI

WT IASFTFPPGMGQFMAGELMPREAISTLFDNNTWVKHVGDPASLGQSAVWI

MUT IASFTFPPGMGQFMAGELMPREAISTLFDNNTWVKHVGDPASLGQSAVWI

451 500

HSap HPRVNVVIIIFLFFVMKFWMSIVATTMPIPCGGFMPVFVLGAAFGRLVGE

WT HPQVNVVIIILLFFIMKFWMSIVATTMPIPCGGFMPVFVLGAAFGRLVGE

MUT HPQVNVVIIILLFFIMKFWMSIVATTMPIPCGGFMPVFVLGAAFGRLVGE

501 550

HSap IMAMLFPDGILFDDIIYKILPGGYAVIGAAALTGAVSHTVSTAVICFELT

WT IMAMLFPDGILFDDIIYKILPGGYAVIGAAALTGAVSHTVSTAVICFELT

MUT IMAMLFPDGILFDDIIYKILPGGYAVI-----------------------

551 600

HSap GQIAHILPMMVAVILANMVAQSLQPSLYDSIIQVKKLPYLPDLGWNQLSK

WT GQIAHILPMMVAVILANMVAQSLQPSLYDSIIQVKKLPYLPDLGWNQLSK

MUT --------------------------------------------------

601 650

HSap YTIFVEDIMVRDVKFVSASYTYGELRTLLQTTTVKTLPLVDSKDSMILLG

WT FTIFVEDIMVRDVKFVSAACTYGELQTLLQTTTVKTLPLVDSKDSMILLG

MUT -------------------------------------------DSMILLG

651 700

HSap SVERSELQALLQRHLCPERRLRAAQEMARKLSELPYDGKARLAGEGLPGA

WT SVERSELQSLLQRHLCPERRLRAAQDMARKLSELPYDGKARPAGRGHQGI

MUT SVERSELQSLLQRHLCPERRLRAAQDMARKLSELPYDGKARPAGRGHQGI

701 750

HSap PP-GRPESFAFVDEDEDEDLSGKSELPPSLALHPSTTAPLSPEEPNGPLP

WT PSEGRRESFAFVDEDEEEDLSGKPELPPLPPPRSFPAALLSPEEPNGPLP

MUT PSEGRRESFAFVDEDEEEDLSGKPELPPLPPPRSFPAALLSPEEPNGPLP

751 800

HSap GHKQQPEAPEPAGQRPSIFQSLLHCLLGRARPTKKKTTQDSTDLVDNMSP

WT SHKQQPEAPEPAGQRPSVFRSLLRCLLGRPRPTKKKTTQESTDLVDTMSP

MUT SHKQQPEAPEPAGQRPSVFRSLLRCLLGRPRPTKKKTTQESTDLVDTMSP

801 850

HSap EEIEAWEQEQLSQPVCFDSCCIDQSPFQLVEQTTLHKTHTLFSLLGLHLA

WT EEIEAWEKEQLSQPVCFDYCCIDQSPFQLVEQTSLHKTHTLFSLLGLHLA

MUT EEIEAWEKEQLSQPVCFDYCCIDQSPFQLVEQTSLHKTHTLFSLLGLHLA

851 900

HSap YVTSMGKLRGVLALEELQKAIEGHTKSGVQLRPPLASFRNTTSTRKSTGA

WT YVTSMGKLRGVLALEELQKAIEGHTKSGVQLRPPLASFRNTTTARKNPGG

MUT YVTSMGKLRGVLALEELQKAIEGHTKSGVQLRPPLASFRNTTTARKNPGG

901 950

HSap PPSSAENWNLPEDRPGATGTGDVIAASPETPVPSPSPEPPLSLAPGKVEG

WT PPPPTDTWSLPEDGTGAAGPGDRAPASPETPVPSPSPEPPLSVAPAKVEG

MUT PPPPTDTWSLPEDGTGAAGPGDRAPASPETPVPSPSPEPPLSVAPAKVEG

951 989

Hsap ELEELELVESPGLEEELADILQGPSLRSTDEEDEDELIL*

WT ELEELELVESPGPEEELADILQGPSLRSTDEEDEDELIL*

MUT ELEELELVESPGPEEELADILQGPSLRSTDEEDEDELIL*

**Figure S1.** ***CLCN1* protein alignment of *Homo sapiens*, domestic cat wildtype (wt) and mutated (mut) protein**. In yellow the three amino acids involved in protein dimerization and in green the two CBS domains. The affected cat protein prediction lacks 116 amino acids, from position 557 to position 643 of the protein sequence.
