# Supplementary material for: A Novel Mutation in CLCN1 Associated with Feline Myotonia Congenita
Source: PLoS One. 2014 Oct 30;9(10):e109926. doi: 10.1371/journal.pone.0109926 (PMC4214686; doi:10.1371/journal.pone.0109926)
Supplement: Figure S2 — Normalized melting curve graph of wild-type, homozygous and heterozygous samples for the c.1930+1G>T polymorphism. The graph represents three different melting curves patterns. The cohort with the highest melting temperature represents the wild type samples, the cohort with the lowest melting curve represents the homozygous affected samples. Samples with a melting curve in between represents the heterozygous group. (DOCX) [file pone.0109926.s002.docx]

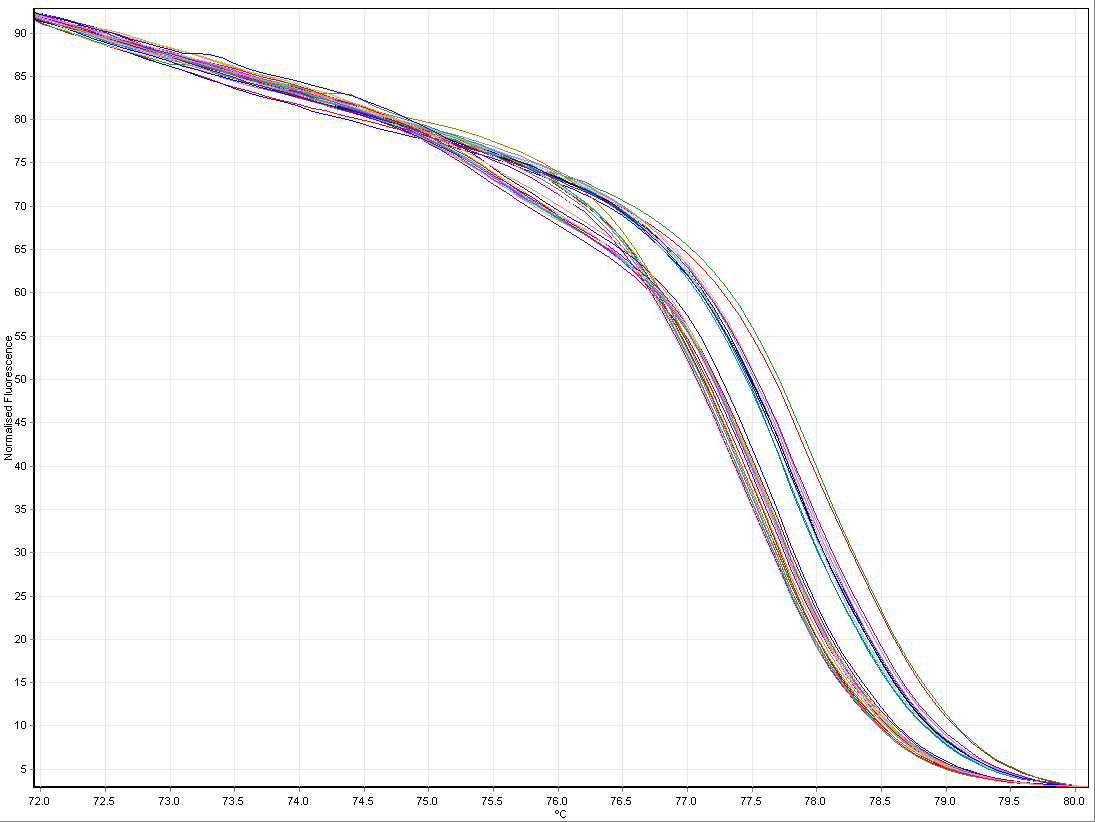


**Figure S2**. Normalized melting curve graph of wild-type, homozygous and heterozygous samples for the c.1930+1G>T polymorphism. The graph represents three different melting curves patterns. The cohort with the highest melting temperature represents the wild type samples, the cohort with the lowest melting curve represents the homozygous affected samples. Samples with a melting curve in between represents the heterozygous group.
