# Supplementary material for: A Novel Mutation in CLCN1 Associated with Feline Myotonia Congenita
Source: PLoS One. 2014 Oct 30;9(10):e109926. doi: 10.1371/journal.pone.0109926 (PMC4214686; doi:10.1371/journal.pone.0109926)
Supplement: Table S1 — Primers to analyze feline CLCN1. (DOCX) [file pone.0109926.s003.docx]

**Table S1. Primers to analyze feline *CLCN1***

| ***CLCN1* Genomic Primers** | | | | | | | | | | |  |
| --- | --- | --- | --- | --- | --- | --- | --- | --- | --- | --- | --- |
| Exon | | Mg^2+^/⁰C | | | Forward Primer 5’-3’ | | | Reverse Primer 5’-3’ | | | |
|  | |  | | |  | | |  | | | |
| 1 | | 1.5/62 | | | TCATGTGACGGAGAGATGGCTATA | | | TACAACACTTCTCCCGCTTTCAC | | | |
| 2-5* | | NA/59 | | | GACCACCACAAAGTGACCCTACAT | | | ACTTCTGTTATTCTGCTCCAGGACTAG | | | |
| 6 | | 1.5/62 | | | TTCTCTAAACCCACATCACACCCT | | | GTGTGTGTGTAGAATAAAGGAGCGTG | | | |
| 7 | | 1.5/62 | | | AAGCCTCTCTTTCTGCCTTATTCC | | | CGGTAAATGCTCACTAAAAGTTTGC | | | |
| 8-12* | | NA/59 | | | AGCAGGTGTATGTTTTAGAGCGTGA | | | TGAATAGACACTGGCTCACTCCTATAGA | | | |
| 13-14 | | 1.5/62 | | | ATGTGTATTGGGCAGGGTTGAG | | | ATGGGAGAGTTTGAGTGTGGCTAT | | | |
| 15-16* | | NA/59 | | | TGTCTCCCCATTCTATGTCACTCC | | | CTAATGACAAGCCCACACTACAGT | | | |
| 17-20* | | NA/59 | | | AGTGCTGAATGAGTGAAATAAAAGGG | | | GCCTGGACTCGCATCTTACTCTTA | | | |
| 21-23* | | NA/59 | | | GCGTATTTCAGGGTCTGGGCT | | | AAACCCCACGGACTATCAGGC | | | |
| ***CLCN1* Sequencing Genomic Primers** | | | | | | | | | | |  |
| Exon | | Primer 5’-3’ | | | |  |  |  |  |  |  |
|  | |  | | | |  |  |  |  |  |  |
| 3 | | GCAGAGATAGACAAGACCCCAGG | | | |  |  |  |  |  |  |
| 4 | | GAGAACAATACCGTGTGGTGAGG | | | |  |  |  |  |  |  |
| 5 | | CTGGAAGCGGCACATAATCACT | | | |  |  |  |  |  |  |
| 9 | | CCAGCAACTCACACTCACCCAC | | | |  |  |  |  |  |  |
| 10 | | GTGGGTGAGTGTGAGTTGCTGG | | | |  |  |  |  |  |  |
| 11 | | GCTGGAGGGATGTCTGTGCTG | | | |  |  |  |  |  |  |
| 18 | | AGAGATTTCCCAGGACACCAGC | | | |  |  |  |  |  |  |
| 19 | | TCAGTTCGCAGAGGCACGC | | | |  |  |  |  |  |  |
| 22 | | TTCTTCCTCCTGCTGCTCCAGT | | | |  |  |  |  |  |  |
| 23 | | GAACCCTCCTCTAAGACTGTCACTATC | | | |  |  |  |  |  |  |
|  | | | **cDNA Primers** | | | | |  |  |  |  |
| Primer name | | | Forward Primer 5’-3’ | | | | |  |  |  |  |
|  | | |  | | | | |  |  |  |  |
| CLCN1- 1F | | | CTTAAGGAGCTGTACTGGGGGAG | | | | |  |  |  |  |
| CLCN1- 1R | | | GAAGAGGATGAGAGTTAGCGGGA | | | | |  |  |  |  |
| CLCN1- 2F | | | GGGACTGCTTATGGCTCTGGTT | | | | |  |  |  |  |
| CLCN1- 2R | | | GACGGCATCCTTGTTCCACAC | | | | |  |  |  |  |
| CLCN1- 3F | | | AACTACTGGCGAGGATTCTTTGC | | | | |  |  |  |  |
| CLCN1- 3R | | | GGAGACCGTATGGGACACTGC | | | | |  |  |  |  |
| CLCN1- 4F | | | TGCTATTCCCTGACGGTATCCTAT | | | | |  |  |  |  |
| CLCN1- 4R | | | GTCCTCCTCTTCATCCTCATCCA | | | | |  |  |  |  |
| CLCN1- 5F | | | ATGGTGCGTGATGTGAAGTTTGT | | | | |  |  |  |  |
| CLCN1- 5R | | | AATCCGTTGACTCCTGGGTTG | | | | |  |  |  |  |
| CLCN1- 6F | | | CAACCCAGGAGTCAACGGATT | | | | |  |  |  |  |
| CLCN1- 6R | | | GTCCTCGTCCTCCTCATCGGT | | | | |  |  |  |  |
| ***CLCN1* HRM Primers** | | | | | | | | | |  |  |
| ⁰C | | | Forward Primer 5’-3’ | | | | Reverse Primer 5’-3’ | | |  |  |
|  | | |  | | | |  | | |  |  |
| 58 | | | AAACCCTGCTCCAGACCACC | | | | TTGTTTCCTTCCTTCCCACTGA | | |  |  |

*Exon acquired using the LongRange PCR kit (Invitrogen) with enhancerA, extension time 1’30” + 10”/cycle
