# Supplementary material for: A Novel Mutation in CLCN1 Associated with Feline Myotonia Congenita
Source: PLoS One. 2014 Oct 30;9(10):e109926. doi: 10.1371/journal.pone.0109926 (PMC4214686; doi:10.1371/journal.pone.0109926)
Supplement: Table S2 — 5′ss exon-intron boundaries strength. (DOCX) [file pone.0109926.s004.docx]

**Table S2.** 5’ss exon-intron boundaries strength.

| Exon-intron | Consensus seq | MaxEnt | MDD | MM | WMM |
| --- | --- | --- | --- | --- | --- |
| 14 | ttggtgaga | 6.29 | 11.68 | 6.58 | 6.27 |
| 15 | caggtcagg | 7.20 | 11.08 | 7.21 | 7.62 |
| 16 | aaggtcagg | 6.23 | 11.68 | 6.65 | 7.25 |
| 16* | aagttcagg | -2.28 | 3.17 | -1.86 | -1.25 |
| 17 | gaggtgacc | 5.57 | 8.48 | 6.10 | 5.43 |

MaxEnt = Maximum Entropy Model score, MDD = Maximum Dependence Decomposition Model score, MM = first-order Markov Model, WMM = Weight Matrix Model score

* Exon-intron strength calculated on the mutated splice site
